# CYP2C19 Contributes to THP-1-Cell-Derived M2 Macrophage Polarization by Producing 11,12- and 14,15-Epoxyeicosatrienoic Acid, Agonists of the PPARγ Receptor

**DOI:** 10.3390/ph16040593

**Published:** 2023-04-15

**Authors:** Hee Young Cho, Sangzin Ahn, Yong-Soon Cho, Su-Kil Seo, Dong Hyun Kim, Jae-Gook Shin, Su-Jun Lee

**Affiliations:** 1 Department of Pharmacology and PharmacoGenomics Research Center, Inje University College of Medicine, Inje University, Busan 47392, Republic of Korea; 2 Center for Personalized Precision Medicine of Tuberculosis, Inje University College of Medicine, Inje University, Busan 47392, Republic of Korea; 3 Department of Microbiology and Immunology, Inje University College of Medicine, Inje University, Busan 47392, Republic of Korea

**Keywords:** cytochrome P450, CYP2C19, M2 macrophage, metabolism, PPAR gamma, EETs

## Abstract

Although the functional roles of M1 and M2 macrophages in the immune response and drug resistance are important, the expression and role of cytochrome P450s (CYPs) in these cells remain largely unknown. Differential expression of the 12 most common CYPs (CYP1A1, 1A2, 1B1, 2B6, 2C8, 2C9, 2C19, 2D6, 2E1, 2J2, 3A4, and 3A5) were screened in THP-1-cell-derived M1 and M2 macrophages using reverse transcription PCR. CYP2C19 was highly expressed in THP-1-cell-derived M2 macrophages, but it was negligibly expressed in THP-1-cell-derived M1 macrophages at the mRNA and protein levels as analyzed by reverse transcription quantitative PCR and Western blot, respectively. CYP2C19 enzyme activity was also very high in THP-1-cell-derived M2 compared to M1 macrophages (> 99%, *p* < 0.01), which was verified using inhibitors of CYP2C19 activity. Endogenous levels of the CYP2C19 metabolites 11,12-epoxyeicosatrienoic acid (11,12-EET) and 14,15-EET were reduced by 40% and 50% in cells treated with the CYP2C19 inhibitor and by 50% and 60% in the culture medium, respectively. Both 11,12-EET and 14,15-EET were identified as PPARγ agonists in an in vitro assay. When THP-1-cell-derived M2 cells were treated with CYP2C19 inhibitors, 11,12- and 14,15-EETs were significantly reduced, and in parallel with the reduction of these CYP2C19 metabolites, the expression of M2 cell marker genes was also significantly decreased (*p* < 0.01). Therefore, it was suggested that CYP2C19 may contribute to M2 cell polarization by producing PPARγ agonists. Further studies are needed to understand the endogenous role of CYP2C19 in M2 macrophages with respect to immunologic function and cell polarization.

## 1. Introduction

Macrophages are dynamic, plastic, and heterogeneous, which means that different control mechanisms and signaling molecules exist for each cell stage. Macrophages have generally been classified into two polarization states (M1 and M2) that express different receptors, cytokines, and chemokines [1]. M1 macrophages exhibit microbicidal and inflammatory properties, whereas M2 macrophages are immunomodulatory cells and exhibit poor microbicidal properties [2]. These findings indicate that activation of macrophages can be pro-inflammatory or anti-inflammatory depending on the intracellular concentration of regulatory molecules. For example, expression of IL-12, CD86, CD40, ICAM-1, IL-1α, IL1β, IL-6, and TNF-α were known to be elevated during polarization of M1 macrophages induced by LPS and interferon-γ in an in vitro system [3]. It has been reported that the expression of TGF-β, IL-10, and CCL18 is elevated during polarization in M2 macrophages induced by IL-4 in an in vitro system [3]. Cytochrome P450 (CYP) enzymes play an important role in drug metabolism and the metabolism of endogenous molecules; however, their expression and function in M1 and M2 cells are largely unknown. Endogenous substrates of CYP enzymes include steroids, fatty acids, bile acid, leukotrienes, and arachidonic acid, which are involved in cardiovascular diseases, immune responses, and inflammatory responses [4]. Metabolism of these endogenous substrates not only removes them from the body but also produces signaling modulators that act as ligands for various types of nuclear receptors, including pregnane X receptor, constitutive androstane receptor, glucocorticoid receptor, aryl hydrocarbon receptor, and peroxisome proliferator-activated receptor (PPAR) [5]. Activation and modulation of these nuclear receptors through differential CYP expression may influence drug metabolism as well as cellular differentiation in M1 and M2 cells. For example, epoxyeicosatrienoic acids (EETs) and 20-hydroxyeicosatetraenoic acid (20-HETE) are CYP-dependent metabolites involved in the regulation of vascular tone and cellular differentiation through the activation of PPARs [6,7,8]. Activation of the PPARγ receptor polarizes macrophages into anti-inflammatory M2 cells and can regulate lipid metabolism [9,10,11,12]. Therefore, we investigated how the 12 most common human CYP genes (CYP1B1, CYP1A1, CYP1A2, CYP2C8, CYP2C9, CYP2C19, CYP2E1, CYP2B6, CYP2D6, CYP2J2, CYP3A4, and CYP3A5) were distributed in THP-1-cell-derived M1 and M2 cells. Metabolism of arachidonic acid by cytochrome P450 can be largely classified into EET and 20-HETE. It is known that EETs are mainly produced by the CYP2 family and that 20-HETE is mainly produced by the CYP4 family [13]. In the present study, when the expression of P450 was screened in THP-1-cell-derived M1 and M2 cells, CYP2C19 was highly expressed in M2 cells. CYP2C19 is also known as a major enzyme synthesizing 11,12-EET and 14,15-EET from arachidonic acid in humans [14]. Therefore, these EETs were selected in this study rather than 20-HETE. In addition, it is known that polarization into M2 cells involves PPARγ activation and expression of genes related to fatty acid metabolism and fat accumulation [9,11,12]. Therefore, we hypothesized that EETs could become an agonist of PPARγ and accelerate the polarization into M2 cells through the expression of PPARγ-dependent genes [8,9,10,11]. The results revealed strong CYP2C19 expression in M2 cells, and elevated levels of 11,12-EET and 14,15-EET were observed along with increased expression of M2 marker genes.

## 2. Results

### 2.1. Cell Differentiation

**Figure 1:**
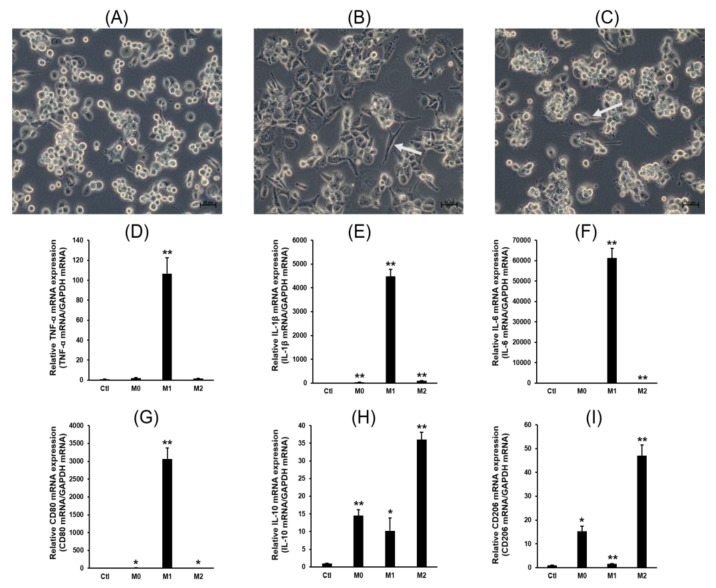
Differentiation of THP-1 monocytes into M1 and M2 macrophages. (**A**) THP-1 cells were induced to form M0 macrophages with the treatment of PMA (200 ng/mL) for 24 h. (**B**) M0 macrophages were polarized into M1 macrophages using 100 ng/mL of LPS and 20 ng/mL of recombinant human IFN-γ. The sharpened shapes of M1 macrophages are indicated by arrow. (**C**) M0 macrophages were polarized into M2 macrophages using 20 ng/mL of recombinant human IL-4 and 20 ng/mL of IL-13. The flattened shapes of M2 macrophages are indicated by arrow. Quantitative real time PCR analysis of M1 and M2 macrophage-specific gene expression was conducted. (**D**) TNF-α mRNA expression, (**E**) IL-1β mRNA expression, (**F**) IL-6 mRNA expression, (**G**) CD80 mRNA expression, (**H**) IL-10 mRNA expression, and (**I**) CD206 mRNA expression. Details of the primers and qRT-PCR conditions are described in the Materials and Methods. Values are presented as the mean ± standard deviation relative to the control (*n* = 3). * *p* < 0.05, ** *p* < 0.01.

Before studying the expression of 12 major CYPs in M1 and M2 macrophages, THP-1 monocytes were induced to form M1 and M2 macrophages, as observed by their cell shape and characteristic gene expression (Figure 1).

### 2.2. Expression Profiles of P450s

**Figure 2:**
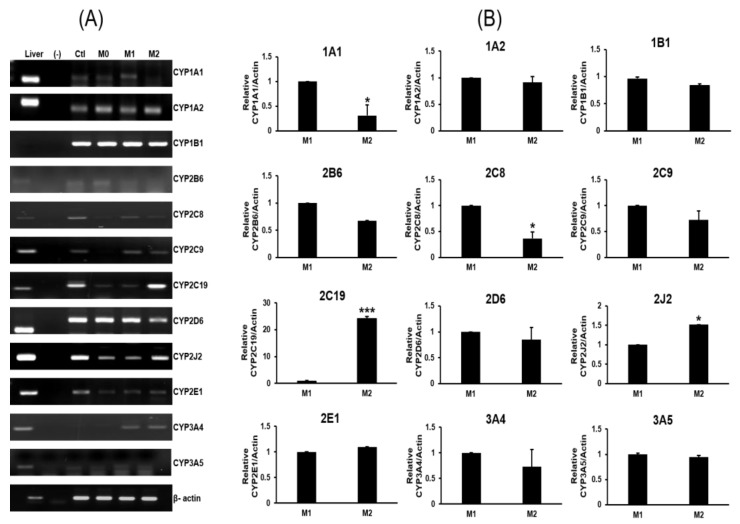
Expression profiles of CYP genes in THP-1 monocytes and M0, M1, and M2 macrophages. (**A**) RT-PCR analysis of CYP expression. RT-PCR was used to amplify the mRNA of each CYP using gene-specific primers as described in the Materials and Methods. Data represent three independent RT-PCR assays. Each target CYP was amplified as a single band with the same size as that found in liver cDNA, which was used as a positive control. No bands were detected in PCR reactions that lacked reverse transcriptase, which confirmed the absence of genomic DNA contamination. (**B**) Quantitative values were determined from three independent experiments using ImageJ software. Values are presented as the mean ± standard deviation relative to β-actin (*n* = 3). * *p* < 0.05, *** *p* < 0.001.

M1 macrophages were induced by IFN-γ/LPS treatment, resulting in the enhanced expression of IL-1β, IL-6, CD80 and tumor necrosis factor alpha (TNF-α). Meanwhile, M2 macrophages were induced by IL-13/IL-4 treatment and displayed upregulated levels of IL-10 and CD206 along with reduced expression of IL-1β, TNF-α, and IL-6. The expression of macrophage-specific CYP genes was investigated using RT-PCR with gene-specific primers (Figure 2).

**Figure 3:**
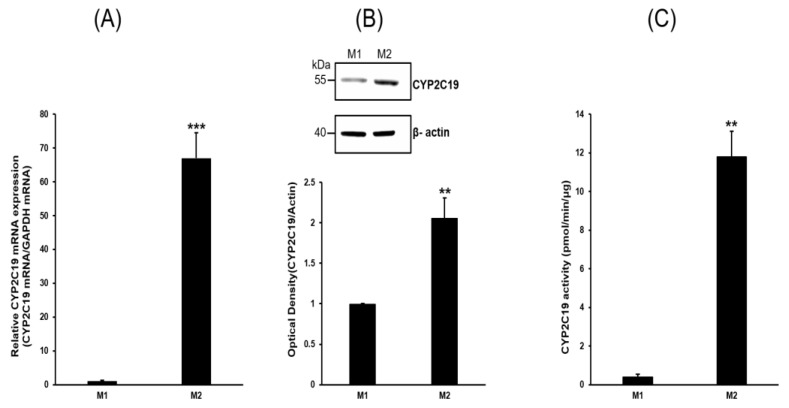
M2-macrophage-specific CYP2C19 expression. (**A**) Expression of CYP2C19 mRNA was determined using RT-qPCR. CYP2C19 mRNA was highly expressed in M2 compared to M1 macrophages. (**B**) Western blot analysis of CYP2C19 and β-actin. CYP2C19 protein expression was high in M2 compared to M1 macrophages. Expression levels were evaluated using a quantitative densitometry method. (**C**) CYP2C19 enzyme activity assay in M1 and M2 macrophages. Membrane fractions of M2 macrophages were incubated with the prototype substrate. The activity was measured using excitation and emission wavelengths of 406 and 468 nm, respectively. Additional details can be found in the Materials and Methods. Values are presented as the mean of three experiments relative to the M1 macrophages. ** *p* < 0.01 and *** *p* < 0.001 versus M1 macrophages.

No detectable bands were observed in the PCR reactions containing RNA samples not treated with reverse transcriptase, and these reactions were used as a control to monitor genomic DNA contamination. A cDNA pool made from human liver (no. 637205; Clontech, Mountain View, CA, USA) was used as a positive control. The following 12 major drug-metabolizing CYPs were screened in THP-1 monocytes, M1 macrophages, and M2 macrophages: CYP1A1, 1A2, 1B1, 2B6, 2C8, 2C9, 2C19, 2D6, 2J2, 2E1, 3A4, and 3A5. Compared to M2 cells, the CYP genes strongly expressed in M1 cells were CYP1A1 and CYP2C8. Compared to M1 cells, the CYP genes strongly expressed in M2 cells were CYP2C19 and CYP2J2. CYP2C19 expression was significantly higher in M2 than M1 cells (*p* < 0.001). The expression of CYP1A2, 1B1, 2B6, 2C9, 2D6, 2E1, 3A4, and 3A5 was not significantly different between M1 and M2 cells. RT-qPCR and Western blot analysis of CYP2C19 confirmed the RT-PCR results in M1 and M2 macrophages; therefore, CYP2C19 protein expression was explored (Figure 3).

### 2.3. Analysis of CYP2C19 Acvitiy and Its Metabolites

**Figure 4:**
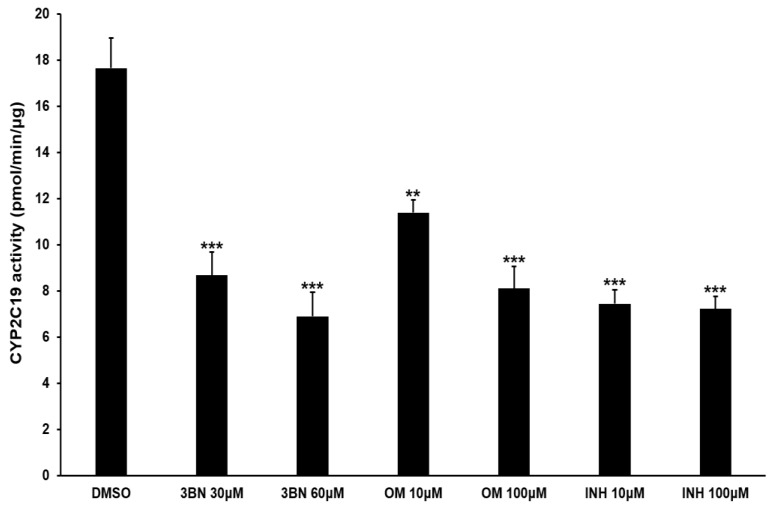
CYP2C19 enzyme activity assay in M2 macrophages. Membrane fractions of M2 macrophages were incubated with the prototype substrate. The activity was measured using excitation and emission wavelengths of 406 and 468 nm, respectively, and was compared with a standard curve. Additional details can be found in the Materials and Methods. Inhibitors of CYP2C19 were used in the reaction to confirm that the reactions were specific to CYP2C19 activity. Data are presented as the mean ± standard deviation of three independent assays. 3BN, (+)-N-3-benzyl-nirvanol; OM, omeprazole; INH, isoniazid. ** *p* < 0.01 and *** *p* < 0.001 versus the vehicle control.

Consistent with the RT-PCR results, CYP2C19 mRNA was highly expressed in M2 compared to M1 macrophages using RT-qPCR analysis (*p* < 0.001). CYP2C19 protein expression more than doubled in M2 compared to M1 macrophages (*p* < 0.01). CYP2C19-specific activity assays were performed in M2 macrophages (Figure 4) and showed a significant reduction (30–60% versus control) with the addition of the selective CYP2C19 inhibitors 3BN, omeprazole, and isoniazid (*p* < 0.01–*p* < 0.001).

**Figure 5:**
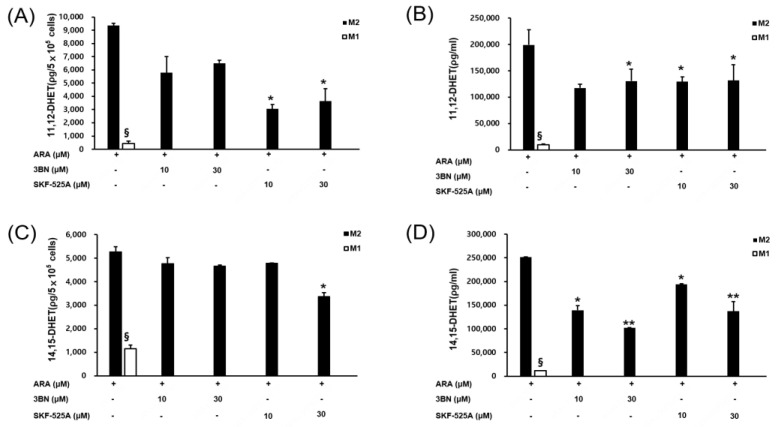
Influence of CYP2C19 inhibitors on 11,12-DHET and 14,15-DHET levels in M2 macrophages. The endogenous CYP2C19 metabolites 11,12-DHET and 14,15-DHET were analyzed in M2 cells treated with the CYP2C19 inhibitor 3BN and the general CYP inhibitor SKF-525A. M2 macrophages were treated with 3BN (10 and 30 µM) and SKF-525A (10 and 30 µM) in the presence of arachidonic acid (ARA; 100 µM) for 24 h. The 11,12-DHET level in the cellular fraction (**A**) and media (**B**). The 14,15-DHET level in the cellular fraction (**C**) and media (**D**). Detailed methods used to determinate metabolite levels are described in the Methods and Materials. ^§^ As a control for comparison with M2 cells, M1 cells were treated with ARA in the same way as M2 cells. Since M1 cells showed very low CYP2C19 enzyme activity and metabolites were not detected after inhibitor treatment, the inhibitor 3BN treatment was omitted in M1 cells. Data are presented as the mean ± standard deviation of three independent experiments. * *p* < 0.05, and ** *p* < 0.01. *** versus the control.

Using the same method, CYP2C19 activity was assessed in THP1 cells, M0 macrophages, and M1 macrophages; however, negligible activity was detected in M0 and M1 cells, and moderate activity was detected in THP1 cells (Figure S1). Therefore, in the experiments evaluating the effect of the CYP2C19 inhibitor on the macrophages, M0 and M1 macrophages were omitted, and only M2 cells, which exhibit CYP2C19 enzyme activity, were treated with the inhibitor and had their activity analyzed. CYP2C19 synthesizes 11,12-EET and 14,15-EET from arachidonic acid. 11,12-EET and 14,15-EET are rapidly hydrolyzed by soluble epoxide hydrolases into 11,12-DHET and 14,15-DHET, which are more stable metabolites that reflect 11,12-EET and 14,15-EET levels [15]. Therefore, CYP2C19 activity was monitored by analyzing these metabolites in M2 macrophages (Figure 5).

Intact M2 macrophages contain very low levels of these metabolites, so arachidonic acid (100 µM) was added to the culture medium before the metabolites were analyzed in the cellular and media fractions. More than 90% of metabolites were detected in the culture media rather than the intracellular fraction, and both metabolites were present in similar amounts in the culture media. The addition of the CYP2C19 inhibitor 3BN to the culture resulted in a 50% reduction in the metabolites. When the epoxygenase inhibitor SKF-525A was added, the activity was inhibited at a level similar to that of 3BN. Treatment of the cells with over 30 µM SKF-525A resulted to cytotoxicity; therefore, this high a concentration was not used subsequently (Figure S2). The media fraction clearly showed the degree of inhibition, but the cellular fraction had relatively low metabolite levels.

### 2.4. PPAR Gamma Activation by CYP2C19 Metabolites and Their Relationship to M2 Marker Gene Expression

**Figure 6:**
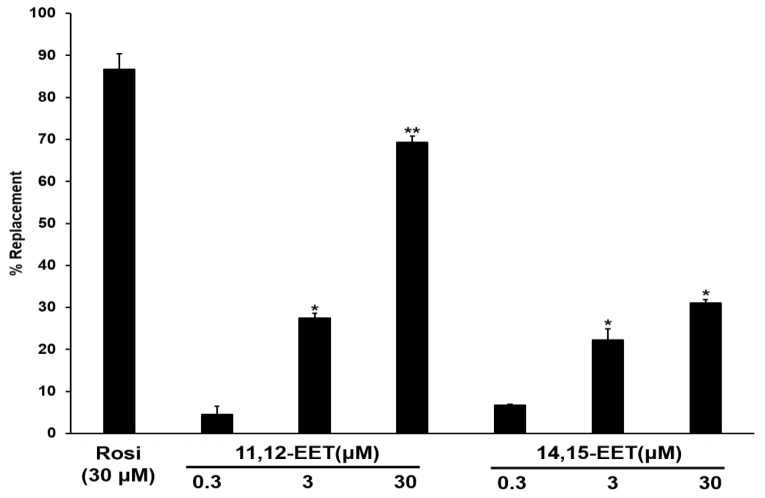
In vitro PPARγ ligand binding assay for 11,12-EET and 14,15-EET. Displacement of the fluorescent PPARγ ligand by 11,12-EET and 14,15-EET was analyzed using an in vitro system and detected by a change in the fluorescence intensity. Rosiglitazone (Rosi), a PPARγ agonist, was used as a positive control. Details are described in the Materials and Methods. All data are presented as the mean ± standard deviation of three independent experiments. * *p* < 0.05, ** *p* < 0.01; linear one-way analysis of variance.

Arachidonic acid metabolites and various fatty acids can act as ligands to PPARγ receptors [16,17]. Therefore, we investigated the ability of 11,12-EET and 14,15-EET to act as PPARγ ligands (Figure 6).

**Figure 7:**
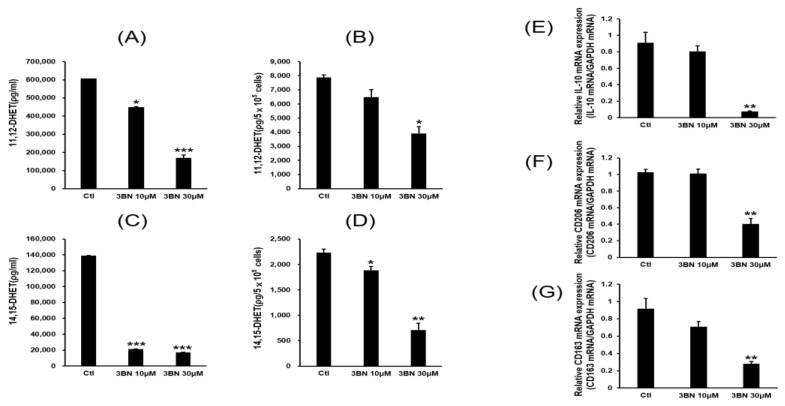
Reduced mRNA expression of selected M2 macrophage marker genes together with a decreased amount of CYP2C19 metabolites by the CYP2C19 inhibitor treatment. The effect of CYP2C19 inhibitor (3BN) on the CYP2C19 metabolites of (**A**) 11,12-DHET in culture media, (**B**) 11,12-DHET in cells, (**C**) 14,15-DHET in culture media, and (**D**) 14,15-DHET in cells. The expression levels of M2 macrophage marker genes were assessed using quantitative real time PCR. (**E**) IL-10 mRNA, (**F**) CD206 mRNA, and (**G**) CD163 mRNA. M2 macrophages were treated with 0, 10, and 30 μM 3BN for 24 h. Metabolite analysis and qRT-PCR were performed as described in the Materials and Methods. Data are expressed as the mean ± standard deviation of triplicate experiments. * *p* < 0.05, ** p < 0.01, and *** *p* < 0.001 versus the control.

As a positive control, rosiglitazone confirmed the accuracy of the method by replacing nearly 90% of the existing fluorescent PPARγ ligand. Both metabolites were shown to be PPARγ ligands in a dose-dependent manner (*p* < 0.05). 11,12-EET was a stronger PPARγ ligand than 14,15-EET. Activation of the PPARγ receptor is involved in the polarization of macrophages into M2 macrophages and also regulates lipid metabolism [9,10,11]. Since CYP2C19 activity produced PPARγ ligands in M2 macrophages, it was inferred that inhibition of CYP2C19 may, in part, reduce PPARγ activity as well as reduce the expression of M2 marker genes. M2 macrophages were treated with the CYP2C19 inhibitor, and an experiment was performed to quantify the metabolites and the expression of the M2 marker genes by quantitative real time PCR in the same sample at the same time (Figure 7). The results showed that the reduction in CYP2C19 activity by the inhibitor in M2 cells reduced the production of 11,12-EET and 14,15-EET metabolites, and these reduced metabolites were accompanied by a decrease in the expression of marker genes in M2 cells.

All marker genes were significantly decreased with inhibitor treatment (*p* < 0.05–0.001). At maximum CYP2C19 inhibition, IL-10 expression was reduced by 90% compared to the control.

## 3. Discussion

Monocyte-derived macrophage cells play an important role in host defense, infection, wound healing, and inflammation. However, studies into the role of CYPs and their expression in these cells are lacking. In hepatocytes, the activity of CYPs, as a drug-metabolizing enzyme system, decreases under conditions that activate immunity. Moreover, LPS, IL-1β, and TNF-α decrease CYP expression in hepatocytes [18,19,20]. CYP activity in macrophages was also reduced with LPS treatment, an immune-activating agent [21]. However, limited information is available regarding the expression of CYPs and their roles in specific cell types, including monocytes and M1 and M2 macrophages. This report is the first to investigate CYP expression and activity by assessing macrophages at each cell stage.

In this study, we differentiated M1 and M2 cells from THP-1 monocytes and examined the expression of the 12 most common CYP genes in humans. The correlation between CYP gene expression and the polarization of macrophage cells was also evaluated. Our findings revealed enhanced CYP2C19 gene expression in M2 compared to M1 macrophages. These results correlated with increased production of 11,12-EET and 14,15-EET as well as the expression of characteristic M2 macrophage genes. The reduction in metabolite levels of 11,12-EET and 14,15-EET upon treatment with CYP2C19 inhibitors indicates they are CYP2C19 specific. M2-specific gene expression was also accompanied by a proportional increase in CYP2C19 activity. The fact that M2 macrophage markers were affected by treatment with CYP2C19-specific inhibitors suggests that the CYP2C19 metabolites 11,12-EET and 14,15-EET could affect M2 macrophage polarization. Among the M2 marker genes in this study, IL-10 was most strongly decreased when the CYP2C19 inhibitor was applied. Therefore, it is presumed that IL-10 is the cytokine most sensitive to CYP2C19 activity. Our results also indicate that 11,12-EET and 14,15-EET metabolites produced by CYP2C19 may induce M2 polarization through activation of PPARγ. Although quantitative real-time PCR results clearly showed that the mRNA expression of the M2 marker genes decreased with CYP2C19 inhibitor treatment, further studies may be needed to confirm the protein levels. We hypothesized that induction of CYP2C19 in M2 cells would increase EETs and that these elevated metabolites would be involved in the increased M2 marker genes. Therefore, studies were conducted to prove this. However, CYP2C19 induction by rifampin and phenobarbital, known as CYP2C19 inducers, did not occur in THP-1-cell-derived M2 macrophages (Figure S3).

Since CYP2J2 was also expressed in THP-1-cell-derived M2 macrophages, its contribution of 11,12-EET and 14,15-EET to M2 differentiation could not be excluded. THP-1-induced M2 macrophages are not capable of siRNA transfection or nucleic acid insertion; therefore, we could not investigate the contribution of CYP2C19 to M2 polarization, which merits further study. Multiple reports indicate M2 macrophage polarization is accelerated in a PPARγ-dependent manner [10,22]. Compared to M1 cells, M2 cells exhibited higher fatty acid metabolism, elevated expression of genes involved in fatty acid metabolism, and enhanced expression of PPARγ [23,24]. PPARγ, an important nuclear receptor involved in regulating genes involved in fatty acid metabolism, is presumed to be activated by the CYP2C19 metabolite and further promotes M2 cell polarization.

In humans, CYP2C19 functions as a major enzyme that produces 11,12-EET and 14,15-EET from arachidonic acid [25]. Our findings demonstrated that 11,12-EET and 14,15-EET activated PPARγ receptors in the assay system. Numerous reports indicate that metabolic deficiency or imbalanced metabolism of arachidonic acid affects cell differentiation and vascular disease [16]. For example, individuals with the CYP2C19 poor metabolizer genotype have been shown to have a higher risk of cardiovascular disease, regardless of the antiplatelet clopidogrel bioactivation [14,26]. This risk is thought to result from the lack of 11,12-EET and 14,15-EET, which act as vasodilators in the vascular system and rely heavily on CYP2C19. Metabolites of arachidonic acid play an important role in cell division and differentiation of gastric mucosal cells, vascular endothelial cells, blood cells, cancer cells, and immune cells [16]. Eicosanoids, metabolites of arachidonic acid, are associated with tuberculosis progression, tuberculosis drug resistance, and tuberculosis drug sensitivity in animal models [27,28,29]. It has been recently reported that 12-HETE, a metabolite of arachidonic acid produced by 12-lipoxygenase, can affect macrophage migration and other pathological progressions in inflammatory diseases [30,31]. Based on our present results, CYP2C19 may, at least in part, contribute to the differentiation process of M2 macrophages and increases the production of anti-inflammatory cytokines in M2 macrophages such as IL-10, thereby suppressing inflammation and limiting tissue damage. In addition to 11,12-EET and 14,15-EET, there are other arachidonic acid metabolites known to be PPARγ agonists, such as 15-HETE, 15-deoxy-∆^12,14^-prostaglandin J_2_ (15d-PGJ_2_), and 13-hydroxyoctadecadienoic acid (13-HODE) [32]. Arachidonic acid metabolism differs between M1 and M2 cells. Xu et al. [33] reported that arachidonic acid metabolism is more active in M2 than M1 cells, and the increase in arachidonic acid metabolism promotes M2 polarization. PPARγ plays an important role in the growth of *Mycobacterium tuberculosis* in macrophages [34]. Interestingly, cytosolic phospholipase A2, which promotes arachidonic acid release [35], also promotes PPARγ activity [36]. The increased PPARγ activation is thought to be caused by enhanced metabolization of free arachidonic acid, which in part produces PPARγ agonists through CYP activity. Therefore, the metabolism of arachidonic acid by CYPs in macrophages likely contributes to the differentiation of M1 and M2 cells. Our findings show that CYP2C19 produces PPARγ agonists in macrophages, which may promote differentiation into M2 cells and play an important role in PPARγ-dependent cell signaling.

However, there are limitations to the present study. It would be useful to determine to what extent the expression of CYP2C19 contributes to M2 polarization among several CYP genes. Further studies are needed to determine whether CYP2C19 expression causes M2 polarization or is a consequence of M2 cell differentiation. It would be important to investigate how blocking CYP2C19 expression during M0 to M2 polarization affects M2 cell full maturation. Additionally, the transcriptional mechanisms underlying why CYP2C19 expression and activity are higher in M2 macrophages compared to M1 cells should be studied. However, targeted siRNA knockdown was not feasible in THP-1-cell-derived macrophages to investigate these phenomena. If CYP2C19 induction by inducers is possible, it would further support our results, but we were unable to test this in M2 macrophages in our experiments. Although we evaluated the expression of M2 marker genes at the mRNA level, a quantitative assessment of the protein levels for these markers would be necessary.

In summary, our results indicate that CYP2C19 is strongly expressed in M2 macrophages. The intrinsic activity of CYP2C19 may contribute to M2 cell polarization during the cell differentiation break point between monocytes and macrophages through the activation of PPARγ signaling by providing its agonists, 11,12- and 14,15-EETs. Strong expression of CYP2C19 in M2 macrophages may also serve as a new biomarker for M2 polarization in the immune environment. The regulatory role of CYP2C19 in M1/M2 macrophage differentiation is important to understanding macrophage-mediated disease progression.

## 4. Materials and Methods

### 4.1. Chemicals and Reagents

Phorbol 12-myristate 13-acetate (PMA), lipopolysaccharide (LPS), sodium dodecyl sulfate (SDS), bovine serum albumin, dimethyl sulfoxide (DMSO), and ethylenediaminetetraacetic acid (EDTA) were purchased from Sigma-Aldrich Chemical Co. (St. Louis, MO, USA). Skim milk was purchased from BD Difco Laboratories, Inc., a subsidiary of Becton, Dickinson, and Company (Sparks, MD, USA). Bradford protein assay reagents and 30% bis-acrylamide solution were purchased from Bio-Rad Laboratories, Inc. (Hercules, CA, USA). All other chemicals and organic solvents used in this study were of the highest grade from commercial sources.

### 4.2. M1 and M2 Cell Culture and Cellular Differentiation

The human leukemic monocyte cell line THP-1 was purchased from the American Type Culture Collection (Manassas, VA, USA). RPMI 1640 medium, fetal bovine serum (FBS), and penicillin–streptomycin were purchased from Hyclone Laboratories, Inc. (Logan, UT, USA). THP-1 cells (ATCC TIB-202) were cultured in RPMI 1640 medium supplemented with 10% FBS, 2-mercaptoethanol (0.05 mM), 100 U/mL of penicillin, and 200 μg/mL of streptomycin at 37 °C in a humidified atmosphere containing 5% CO_2_. THP-1 cells were distributed (2.0 × 10^5^ cells/dish) in a T75 cell culture flask. Differentiation of THP-1 cells into macrophage-like cells (M0) was induced with treatment of 200 ng/mL of PMA for 24 h. M0 cells polarized into M1 macrophage cells with treatment of 100 ng/mL of LPS and 20 ng/mL of recombinant human interferon gamma (IFN-γ; Catalog number 285-IF-100; R&D Systems, Minneapolis, MN, USA). M2 macrophages were selected for using 20 ng/mL recombinant human interleukin 4 (IL-4; Catalog number 200-04; PeproTech, Rocky Hill, NJ, USA) and 20 ng/mL of recombinant human IL-13 (Catalog number 200-13-50; PeproTech) as previously described [37]. Chemicals used to treat the cell culture were dissolved in DMSO, and vehicle-treated cells received an equivalent volume of DMSO (<0.01% of total media volume) as the control. Cellular differentiation was confirmed using gene-specific PCR and cell morphology, as previously reported [37,38]. PCR primers of the characteristic genes associated with each cell type are described in Table 1. Optic phase-contrast photographs were taken to confirm cell differentiation using a 20×/0.25 NA objective lens on an Olympus CKX41 inverted microscope (Olympus Life Science, Tokyo, Japan).

### 4.3. RNA Extraction and Reverse Transcription Polymerase Chain Reaction (RT-PCR)

Total cellular RNA was isolated using TRIzol reagent (Invitrogen, Carlsbad, CA, USA) according to the manufacturer′s instructions. Each RNA was quantified and qualified using a NanoDrop 2000 spectrophotometer (Thermo Fisher Scientific, Waltham, MA, USA). Total RNA (1 µg) was reverse-transcribed into cDNA using an iScript cDNA synthesis kit (Bio-rad, CA, USA). Briefly, a reaction mixture containing 100 pmol oligo (dT)18, 2.5 mM dNTP, and 200 U of M-MLV reverse-transcriptase was incubated for 5 min at 25 °C and subsequently for 20 min at 46 °C. The reaction was terminated by elevating the temperature to 95 °C for 1 min. The specific CYP primers were designed to amplify the target gene based on reference papers. The amplified P450 gene and references include CYP1A1 [40]; CYP1B1 [39]; CYP1A2, CYP2B6, CYP2C8, CYP2C9, CYP2C19, CYP2D6, CYP2E1, CYP3A4, and CYP3A5 [41]; and CYP2J2 [42]. Synthesized cDNA was subjected to PCR amplification in a 30 μL reaction volume containing 10 mM dNTPs, 25 mM MgCl_2_, 10 pmol of both the forward and reverse primers (Table 1), and 1 U of Ex-Taq DNA polymerase (Takara Bio Inc., Otsu, Japan). PCR reactions were performed as follows: initial denaturation at 95 °C for 3 min; 35 cycles of denaturation at 95 °C for 30 s, annealing at 60 °C for 40 s, elongation at 72 °C for 40 s; and a final cycle of elongation at 72 °C for 7 min. Amplified DNA was separated on a 1.5% (*w/v*) agarose gel and stained with ethidium bromide. Reverse transcription quantitative PCR (RT-qPCR) was performed using primer and probe sets for GAPDH (catalogue number Hs02786624_m1), CYP2C19 (catalogue number Hs00426380_m1) acquired from Applied Biosystems, TNF-α (catalogue number Hs00426380_m1), IL-1β (catalogue number Hs01555410_m1), IL-6 (catalogue number Hs00174131_m1), IL-10 (catalogue number Hs00961622_m1), CD80 (catalogue number Hs01045161_m1), and CD163 (catalogue numberHs00174705_m1) (Foster City, CA, USA). The reactions were performed using an ABI Prism 7900 Sequence Detector (Applied Biosystems). Relative mRNA expression was calculated using the difference between the Ct value of GAPDH mRNA and the target mRNA (∆Ct method), as previously described [45]. The results are presented as the ratio of target mRNA to GAPDH mRNA (2^−(∆∆Ct)^). The assays were performed twice with independent triplicates, and the results are presented as the mean ± standard error.

### 4.4. Protein Extraction and Immunoblot Assay

Cells were harvested and washed twice with cold phosphate-buffered saline (PBS). Total protein was extracted using RIPA lysis buffer (pH 7.4) that contained 0.5% SDS, 1% Nonidet P-40, 1% sodium deoxycholate, and protease inhibitors. Protein concentrations were determined using the Bio-Rad Protein Assay Dye reagent (Bio-Rad, Hercules, CA, USA). Proteins (30 µg/lane) were separated by electrophoresis in a 10% Mini-PROTEAN^®^ TGX™ Precast Gel (Bio-rad CA, USA) and transferred to a polyvinylidene fluoride membrane (GE Healthcare Bio-Sciences, Piscataway, NJ, USA). The membranes were blocked using 5% skim milk in Tris-buffered saline containing 0.01% Tween 20 (T-TBS). The membrane was then incubated with specific primary antibodies overnight at 4 °C. Specific anti-CYP2C19 antibodies (catalog number, ab137015) (Abcam, Cambridge, UK) were used at a 1:1000 dilution, along with β-actin at a dilution of 1:1000 (Santa Cruz Biotechnology, Dallas, TX, USA). After washing with T-TBS three times, the membrane was incubated with horseradish-peroxidase-conjugated secondary antibodies for 1 h at room temperature. Images were captured using a ChemiDoc Imaging System (Bio-rad, CA, USA).

### 4.5. CYP2C19 Enzyme Activity Assay

The cells were harvested, washed with cold phosphate-buffered saline, and lysed in homogenization buffer (0.1 M potassium phosphate buffer (pH 7.4), 1 mM EDTA, 1 mM dithiothreitol, 0.1 mM phenylmethylsulfonyl fluoride, and 20% glycerol). The cell lysates were centrifugated at 100,000× *g* for 60 min to separate membrane fractions from soluble fractions. The membrane fraction was resuspended in buffer (0.1 M potassium phosphate buffer (pH 7.4), 1 mM EDTA, and 20% glycerol) and stored at −80 °C until analysis, as previously reported [46]. CYP2C19 enzyme activity was measured according to the manufacturer’s instructions (CYP2C19 Activity Assay Kit, Catalog number ab 211072; Abcam). Briefly, the reaction included membrane protein (300 µg), various concentrations of CYP2C19 inhibitor ((+)-N-3-benzylnirvanol; 3BN), and the assay buffer (NADPH-generating reagent, nonfluorescent CYP2C19 substrate). The fluorescent CYP2C19 metabolite was detected at excitation/emission 406/468 nm on a microplate reader in kinetic mode for 60 min at 37 °C using a microplate reader (Molecular Devices, San Jose, CA, USA). Background activity was normalized using a reaction mixture that did not contain membrane protein.

### 4.6. EET Assays

An enzyme-linked immunosorbent assay (ELISA) was used to measure 14,15-DHET and 11,12-dihydroxyeicosatrienoic acid (11,12-DHET) according to the manual (14,15-DHET ELISA kit; Catalog number DH4 and DH1; Detroit R&D Inc., Detroit, MI, USA). Briefly, cell cultures were harvested and centrifuged at 900× *g* for 3 min to isolate the cell pellet. Manufacturer-provided extraction buffer (pH 3) was added to the sample along with an equal volume of ethyl acetate (250 µL). After aggressive vortex mixing and centrifugation, the organic phase was collected. Collected extracts were evaporated under nitrogen, and dried extracts were eluted in ethanol. DHET in ethanol was measured according to the manufacturer’s instructions.

### 4.7. Binding Analysis of 11,12-EET and 14,15-EET to PPARγ

The PPARγ Ligand Screening Assay Kit (BioVision, Milpitas, CA, USA) was used to determine if 11,12-EET and 14,15-EET bind to PPARγ. Both 11,12-EET and 14,15-EET were purchased from Sigma-Aldrich. The assay provides a single-step fluorescence-based method for identifying potential PPARγ-specific ligands. The assay uses the ability of 11,12-EET and 14,15-EET to displace a fluorescent probe, resulting in the loss of probe fluorescence. Rosiglitazone (Sigma-Aldrich) was used as a positive control [38,47].

### 4.8. Cell Viability Assay

The cytotoxicity of the chemicals was determined by using a Cell Counting Kit-8 (CCK-8) assay (Dojindo Lab, Kumamoto, Japan) [48]. Briefly, cells were grown in 96-well plates for 24–48 h while treated with chemicals. Cells were then washed, and the CCK solution (10 µL) was added to each well, followed by 1 h incubation at 37 °C in a humidified atmosphere containing 5% CO_2_. The amount of formazan dye generated was determined by measuring absorbance at 450 nm using a microplate reader (Molecular Devices). Cell viability was expressed as a percentage of the untreated control cells.

### 4.9. Data Analysis

Data were analyzed using a Student’s t-test and analysis of variance with SigmaPlot (version 8.0, SPSS Inc., Chicago, IL, USA). All data are presented as means ± S.D. of three independent experiments. Statistically significant changes compared with the control group were indicated as * *p* < 0.05, ** *p* < 0.01, and *** *p* < 0.001.

## Figures and Tables

**Table 1:** Primers for RT-PCR.

Gene and Primer ^a^	Sequence (5′→3′)	References
CYP1B1-FP CYP1B1-RP	AACTGTCCATCAGGTGAGGT TAAGGAAGTATACCAGAAGGC	[39]
CYP1A1-FP CYP1A1-RP	CTTCATCCTGGAGACCTTCC AAGACCTCCCAGCGGGCAA	[40]
CYP1A2-FP CYP1A2-RP	GGAGGCCTTCATCCTGGAGA TCTCCCACTTGGCCAGGACT	[41]
CYP2C8-FP CYP2C8-RP	TTCATGCCTTTCTCAGCAGG ATTTGTGCAGTGACCTGAAC	[41]
CYP2C9-FP CYP2C9-RP	TTCATGCCTTTCTCAGCAGG TTGCACAGTGAAACATAGGA	[41]
CYP2C19-FP CYP2C19-RP	TTCATGCCTTTCTCAGCAGG ACAGATAGTGAAATTTGGAC	[41]
CYP2E1-FP CYP2E1-RP	TGCCATCAAGGATAGGCAAG AATGCTGCAAAATGGCACAC	[41]
CYP2B6-FP CYP2B6-RP	ACACAGTGAATTCAGCCACC TGGTGTGTTGGGTGACAATG	[41]
CYP2D6-FP CYP2D6-RP	CCTGTGCATAGTGGTGGCTG GCTTCTCCCAGACGGCCTCA	[41]
CYP2J2-FP CYP2J2-RP	GGACTCTCCTACTGGGCACT CTCCGA AGGTGATGGAGCAA	[42]
CYP3A4-FP CYP3A4-RP	TGACCCAAAGTACTGGACAG CTATTCACAAAGTAATTTGAGG	[41]
CYP3A5-FP CYP3A5-RP	TGACCCAAAGTACTGGACAG TGAAGAAGTCCTTGCGTGTC	[41]
GAPDH-FP GAPDH-RP	AATGCATCCTGCACCACCAA GTAGCCATATTC ATTGTCAT	[43]
β-actin-FP β-actin-RP	GGCGGCACCACCATGTACCCT AGGGGCCGGACTCGTCATACT	[44]

^a^ FP, forward primer; RP, reverse primer.

## Data Availability

Data is contained within the article and Supplementary Material.

## Supplementary Materials

The following supporting information can be downloaded at: https://www.mdpi.com/article/10.3390/ph16040593/s1, Figure S1: Comparative analysis of CYP2C19 activity in THP-1 cells, THP-1-derived M0, M1, and M2 macrophages. Figure S2: Viability of THP1-derived M2 macrophages. Figure S3: Western blot analysis of CYP2C19 proteins in THP1-derived M2 macrophages treated with rifampin and phenobarbital.
