# Supplementary material for: CYP2C19 Contributes to THP-1-Cell-Derived M2 Macrophage Polarization by Producing 11,12- and 14,15-Epoxyeicosatrienoic Acid, Agonists of the PPARγ Receptor
Source: Pharmaceuticals (Basel). 2023 Apr 15;16(4):593. doi: 10.3390/ph16040593 (PMC10143178; doi:10.3390/ph16040593)
Supplement: Supplementary file 1 [file pharmaceuticals-16-00593-s001.zip › pharmaceuticals-2328460-supplementary.pptx]

## Slide 1
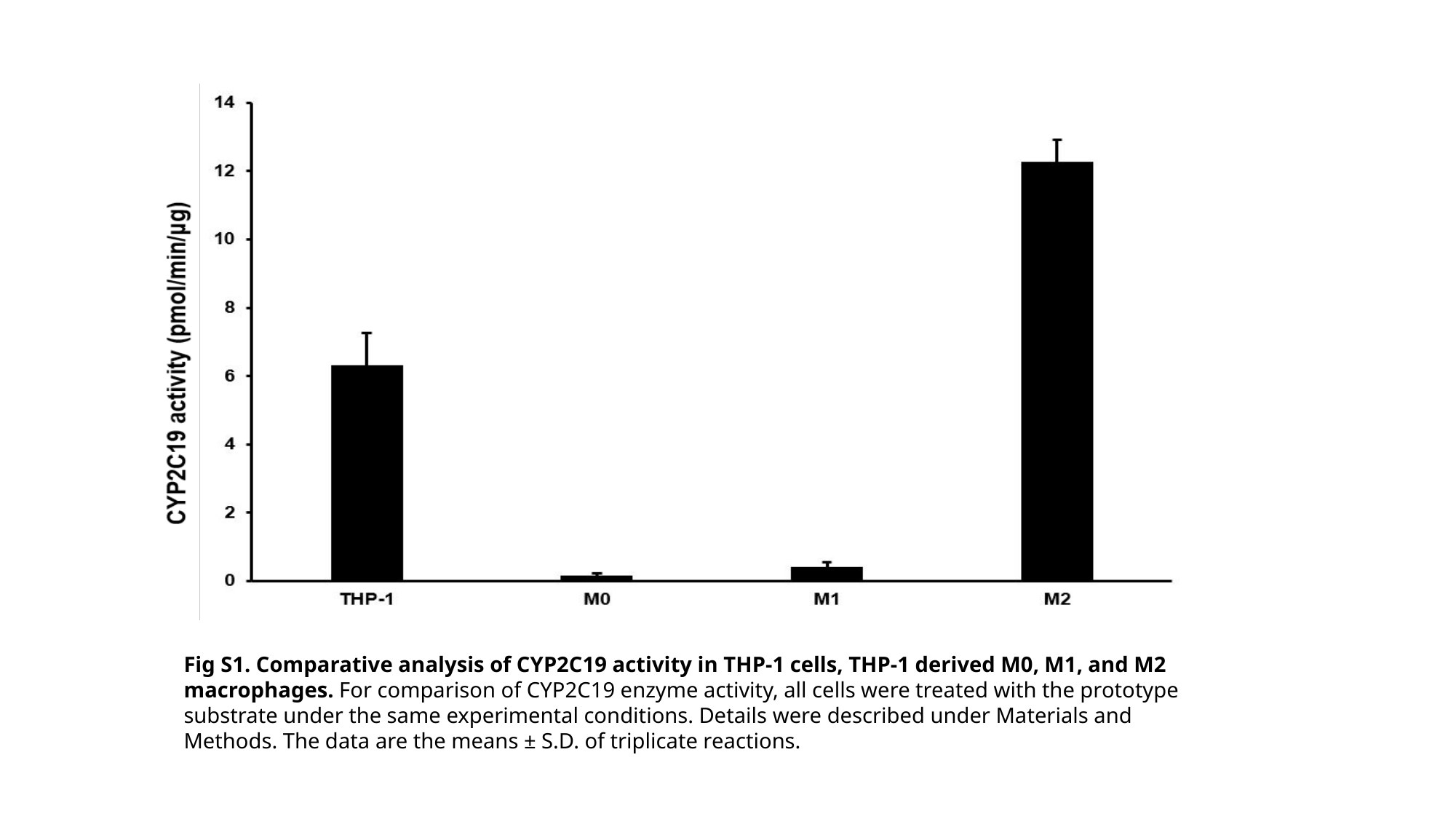

Fig S1. Comparative analysis of CYP2C19 activity in THP-1 cells, THP-1 derived M0, M1, and M2 macrophages. For comparison of CYP2C19 enzyme activity, all cells were treated with the prototype substrate under the same experimental conditions. Details were described under Materials and Methods. The data are the means ± S.D. of triplicate reactions.

## Slide 2
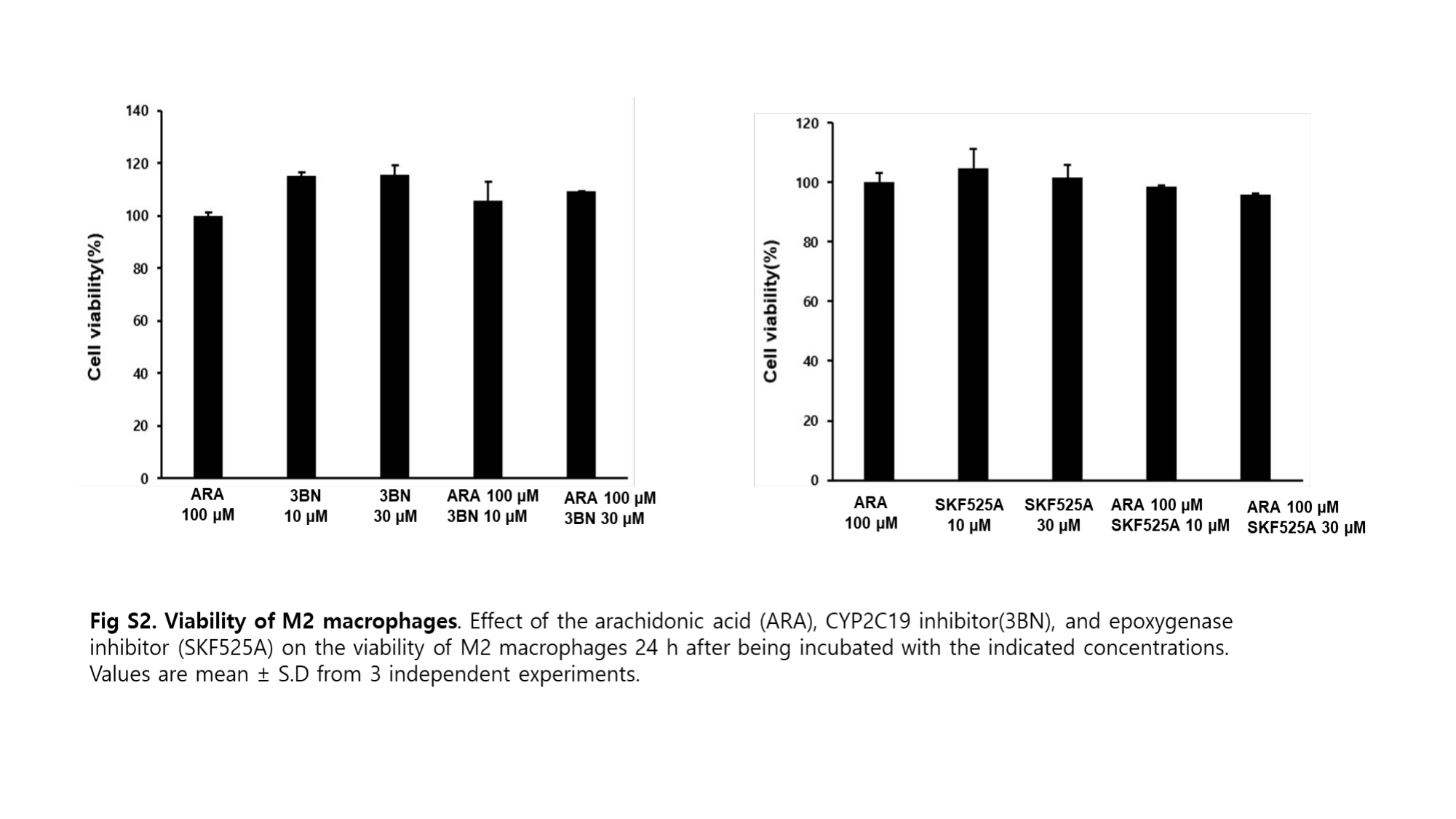

## Slide 3
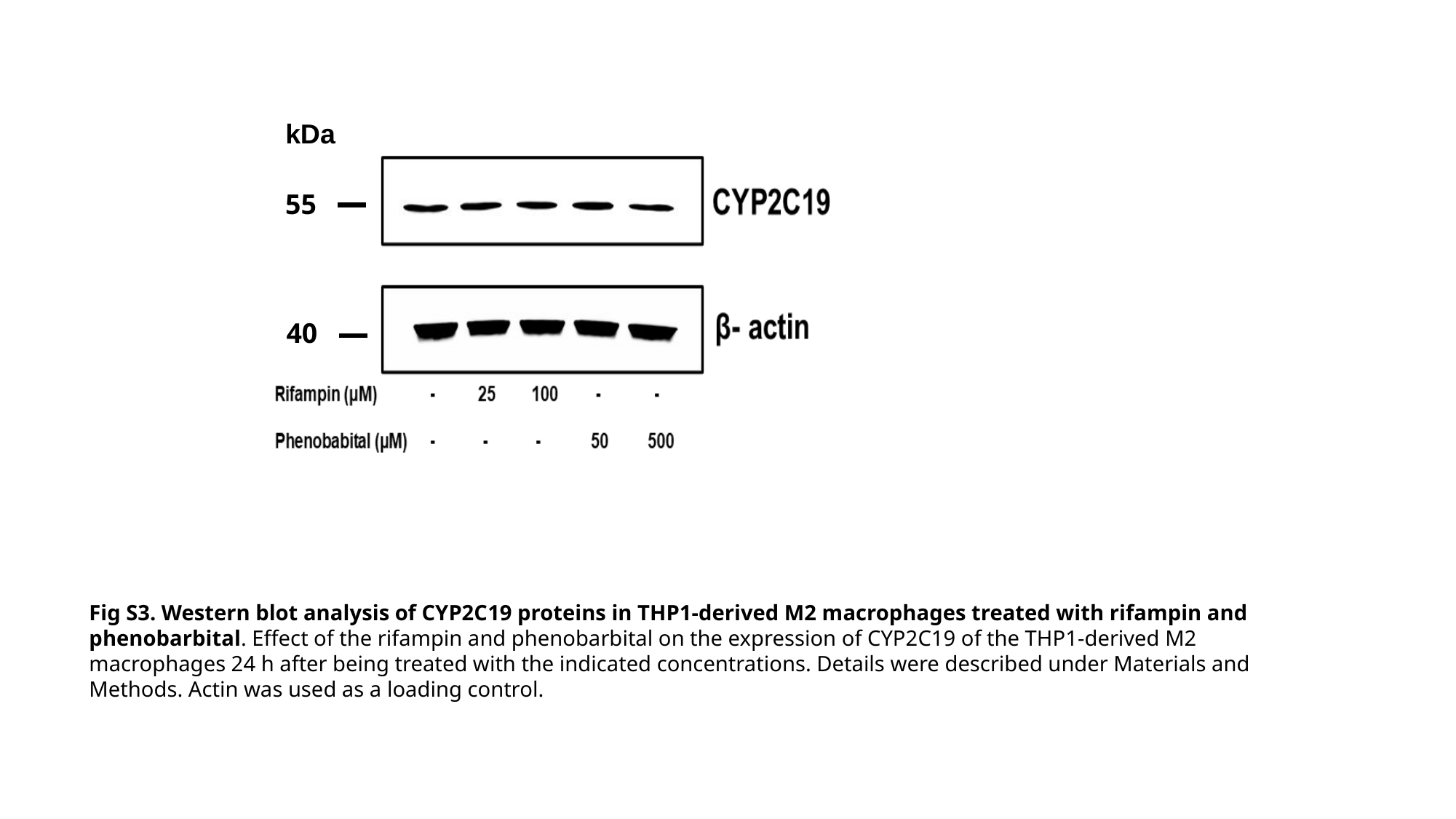

kDa
55
40
Fig S3. Western blot analysis of CYP2C19 proteins in THP1-derived M2 macrophages treated with rifampin and phenobarbital. Effect of the rifampin and phenobarbital on the expression of CYP2C19 of the THP1-derived M2 macrophages 24 h after being treated with the indicated concentrations. Details were described under Materials and Methods. Actin was used as a loading control.
